# Association between ideal cardiovascular health and abnormal glucose metabolism in the elderly: evidence based on real-world data

**DOI:** 10.1186/s12877-023-04632-4

**Published:** 2024-05-10

**Authors:** Yongcheng Ren, Wenwen Wang, Haiyin Zou, Yicun Lei, Yiduo Li, Zheng Li, Xiaofang Zhang, Lingzhen Kong, Lei Yang, Fuqun Cao, Wei Yan, Pengfei Wang

**Affiliations:** 1 Affiliated Hospital of Huanghuai University, Zhumadian Central Hospital, Zhumadian, 463000 He’nan People’s Republic of China; 2 Institute of Health Data Management, Huanghuai University, Zhumadian, 463000 He’nan People’s Republic of China; 3 Digital Medicine Center, Pingyu People’s Hospital, Zhumadian, He’nan People’s Republic of China; 4 Department of Chronic Disease Prevention and Control, Center for Disease Control and Prevention, Jiyuan, 459099 He’nan People’s Republic of China

**Keywords:** Cardiovascular health, Elderly, Abnormal glucose metabolism

## Abstract

**Background:**

Limited information is available on the effect of ideal cardiovascular health (CVH) and abnormal glucose metabolism in elderly people. We aimed to analyze the prevalence of CVH behaviors, abnormal glucose metabolism, and their correlation in 65 and older people.

**Methods:**

In this study, randomized cluster sampling, multivariate logistic regression, and mediating effects analysis were used. Recruiting was carried out between January 2020 and December 2020, and 1984 participants aged 65 years or older completed the study.

**Results:**

The prevalence of abnormal glucose metabolism in this group was 26.7% (*n* = 529), among which the prevalence of impaired fasting glucose (IFG) was 9.5% (male vs. female: 8.7% vs 10.1%, *P* = 0.338), and the prevalence of type 2 diabetes mellitus (T2DM) was 19.0% (male vs. female: 17.8 vs. 19.8%, *P* = 0.256). The ideal CVH rate (number of ideal CVH metrics ≥ 5) was only 21.0%. The risk of IFG and T2DM decreased by 23% and 20% with each increase in one ideal CVH metrics, with OR (95%CI) of 0.77(0.65–0.92) and 0.80(0.71–0.90), respectively (*P*_-trend_ < 0.001). TyG fully mediated the ideal CVH and the incidence of T2DM, and its mediating effect OR (95%CI) was 0.88(0.84–0.91).

**Conclusions:**

Each increase in an ideal CVH measure may effectively reduce the risk of abnormal glucose metabolism by more than 20%.

**Supplementary Information:**

The online version contains supplementary material available at 10.1186/s12877-023-04632-4.

## Introduction

Abnormal glucose metabolism and aging have become one of the main public health issues worldwide [1, 2]. Preventing and managing of abnormal glucose metabolism, which is in increasing demand in the elderly population, requires further research and optimization [3], the prevalence of IFG or/and HbA1c prediabetes even reached 53% in the Asia data-based high-risk population [4]. In 2010, the American Heart Association proposed four intervenable factors (smoking, body mass index, diet, physical activity) and three physiological factors (blood pressure, total cholesterol, fasting plasma glucose) to distinguish different levels of cardiovascular health (CVH). With the aim of “improving the CVH level of the population and reducing the risk of cardiovascular events”, CVH has made good progress in different cardiovascular events, including myocardial infarction, stroke, coronary heart disease, heart failure, sudden cardiac death, angina pectoris, and all-cause death [5–7]. However, there are relatively few studies on the association between CVH levels and abnormal glucose metabolism in Asia, mainly in European and American populations. There is only one cohort study in China, which is based on different occupational groups [8]. It is also unclear whether there are mediating variables in the correlation of CVH level and the incidence of abnormal glucose metabolism, and there is lack of research evidence in elderly groups older than 65 years. Therefore, this study analyzed CVH levels, the prevalence of abnormal glucose metabolism, and their correlation in elderly population over 65 years of age, as well as provided evidence based on natural population for prevention and management strategies for elderly groups with abnormal glucose metabolism.

## Materials and methods

### Sampling and study population

**Fig. 1:**
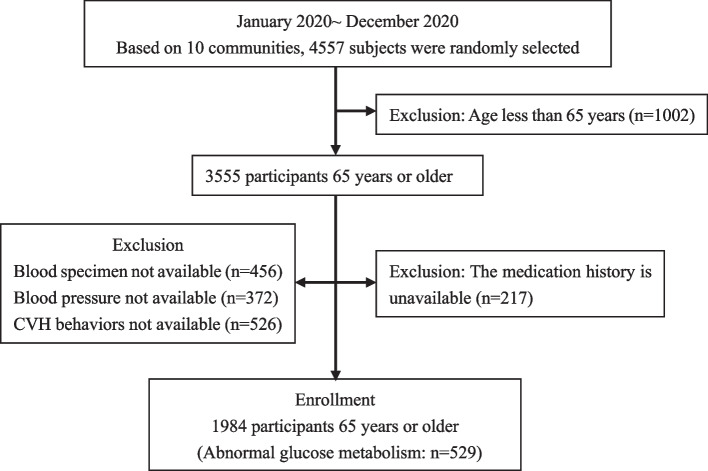
Study flow. CVH- cardiovascular health

The health record information of permanent residents 65 years or older in 2020 was selected for this study using a random cluster sampling method during January-December 2022, with the local community health service center as the main unit. A total of 1,984 subjects (529 abnormal glucose metabolism) were included in the study, as shown in Fig. 1.

### Main research content

The study included demographic characteristics: age, gender, occupation, ethnicity, education level and marital status; Behavioral risk factors: smoking history, drinking history, physical activity, dietary habits; Family history of disease: family history of hypertension, diabetes and other cardiovascular and cerebrovascular diseases; Anthropometric parameters: height, weight, waist circumference; Blood pressure, heart rate; Biochemical indicators: total cholesterol, triglyceride, low density lipoprotein, high density lipoprotein, fasting plasma glucose; Medication history.

### Definitions of key variables

Based on the China Guidelines for the Diagnosis and Treatment of Diabetes in the Elderly (2021 edition), the diagnostic criteria for type 2 diabetes were chosen as the following: typical symptoms of diabetes plus fasting venous plasma glucose ≥ 7.0 mmol/L, or diagnosed by a doctor and receiving medication; The diagnostic criteria for impaired fasting plasma glucose were: fasting plasma glucose ≥ 6.1 mmol/L and < 7 mmol/L without drug intervention. Triglyceride glucose index (TyG): a marker of insulin resistance, calculated as TyG = Ln (TG × FPG/2) (both TG and FPG have mg/dL as a unit). Based on the China Guidelines for the Prevention and Treatment of dyslipidemia in adults (2016 edition), the diagnostic criteria for metabolic syndrome include any three or more abnormalities: (1) male (WC > 90 cm), female (WC > 85 cm); (2) TG ≥ 1.7 mmol/L; (3) HDL-C < 1.0 mmol/L; (4) SBP ≥ 130 or DBP ≥ 85 mmHg or with a history of hypertension; (5) FPG ≥ 6. l mmol/L. The diagnostic criteria for hypertension are SBP ≥ 140 or DBP ≥ 90 mmHg or a history of hypertension. Ideal CVH behavior is defined as (1) Ideal smoking: the number of cigarettes used does not exceed 100 at the time of the survey date; (2) Ideal BMI: < 24 kg/m^2^; (3) Ideal diet: reasonable mix of meat and vegetables, non-high-fat, high-salt diet; (4) Ideal physical activity: light physical activity for more than 30 min and/or moderate physical activity for more than 20 min and/or heavy physical activity for more than 10 min every day; (5) Ideal TC level: < 5.18 mmol/L. Because abnormal glucose metabolism was diagnosed by fasting plasma glucose (FPG) as the outcome event of this study, the ideal CVH index in this study did not include FPG.

### Statistical analysis

**Fig. 2:**
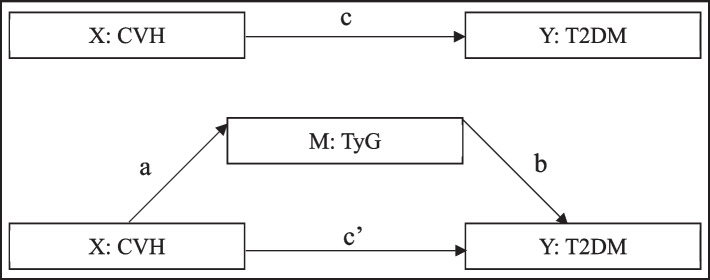
Mediating pathway diagram of the relationship between TyG level in CVH and the incidence of T2DM. CVH- cardiovascular health, T2DM- type 2 diabetes mellitus, TyG- Triglyceride glycemic index

Epi-Data 3.1 was used to establish and manage the database and passed the consistency test. Categorical variables were described by percentage and χ2 test was used to analyze differences between groups. Continuous variables were described by mean ± standard deviation and analyzed by t test. The logistic regression model was used to analyze the association between ideal CVH metrics and abnormal glucose metabolism, and the OR and its 95% CI were used to describe the association (adjusted factors included age, sex, occupation, heart rate, family history of diabetes, smoking, and alcohol consumption). PROCESS was used to analyze the mediation effect between theCVH level mediated by TyG and T2DM, with the CVH level as the independent variable (X), T2DM as the dependent variable (Y), and the TyG as the mediator variable (M). The relationship among the three is shown in Fig. 2, where path C represents the total effect, that is, when TyG is not considered, the total effect of CVH level on the incidence of T2DM; Path C' represents the direct effect, that is, the effect of CVH level on the incidence of T2DM after TyG is included; Path ab represents the indirect effect (mediating effect), that is, the effect of CVH level on the incidence of T2DM through TyG. The mediating effect values and the 95% CI were calculated by random sampling for 10000 times using nonparametric percentile bootstrap method with bias correction. All analyzes were performed using SPSS 23.0 statistical software, with a two-sided test level of α = 0.05.

## Results

### Basic characteristics of the participants

A total of 1984 subjects were included in the study, with an age composition of 73.39 ± 5.89 years, of which 865 (43.6%) were males and 1119 (56.4%) were females. Differences in ideal physical activity, BMI level, and DBP level in ideal CVH metrics were not statistically significant (*P* > 0.05) compared between males and females. The overall prevalence of abnormal glucose metabolism was 26.7% (529/1984), with a 9.5% prevalence of IFG (male vs. female: 8.7% vs. 10.1%, *P* = 0.338); and a 19.0% prevalence of T2DM (male vs female: 17.8 vs. 19.8%, *P* = 0.256) (Table 1).

**Table 1:** Basic characteristics of the objects

Basic characteristics	total (*n* = 1984)	Male (*n* = 865)	Female (*n* = 1119)	*P* value^*^
Age(year)	73.39 ± 5.89	73.20 ± 5.60	73.55 ± 6.10	0.185
High school and above education (%)	459 (25.4)	231 (29.4)	228 (22.4)	0.001
Married and living together (%)	1746 (88.0)	780 (90.3)	966 (86.3)	0.007
Ideal smoking status (%)	1852 (93.9)	748 (86.9)	1104 (99.4)	< 0.001
Drinking (%)	148 (7.5)	143 (16.6)	5 (0.40)	< 0.001
Ideal healthy diet (%)	1898 (95.7)	836 (96.8)	1062 (94.9)	0.043
Ideal physical activity (%)	572 (28.8)	258 (29.9)	314 (28.1)	0.380
Family history of diabetes (%)	41 (2.1)	20 (2.3)	21 (1.9)	0.497
IFG (%)	153 (9.5)	62 (8.7)	91 (10.1)	0.338
T2DM (%)	376 (19.0)	154 (17.8)	222 (19.8)	0.256
AGM (%)	529 (26.7)	216 (25.0)	313 (28.0)	0.134
HTN (%)	733 (36.9)	333 (38.5)	400 (35.7)	0.201
MS (%)	446 (22.5)	152 (17.7)	294 (22.6)	< 0.001
HR (times/min)	75.79 ± 11.20	75.33 ± 11.56	76.15 ± 10.91	0.108
BMI (kg/m^2^)	24.94 ± 3.37	24.83 ± 3.23	25.03 ± 3.48	0.196
SBP (mmHg)	140.66 ± 18.72	138.95 ± 17.76	141.97 ± 19.34	< 0.001
DBP (mmHg)	79.34 ± 12.29	79.63 ± 11.85	79.11 ± 12.63	0.350
FPG (mmol/L)	5.52 ± 1.62	5.51 ± 1.63	5.53 ± 1.61	0.778
TC (mmol/L)	5.24 ± 1.71	4.98 ± 1.48	5.45 ± 1.84	< 0.001
TG (mmol/L)	1.99 ± 1.28	1.82 ± 1.21	2.12 ± 1.32	< 0.001
HDL-C (mmol/L)	1.39 ± 0.38	1.33 ± 0.34	1.44 ± 0.40	< 0.001
LDL-C (mmol/L)	3.12 ± 0.89	2.98 ± 0.83	3.23 ± 0.92	< 0.001

*IFG* Impaired fasting glucose, *T2DM* Type 2 diabetes mellitus, *AGM* Abnormal glucose metabolism, *HTN* Hypertension, *MS* Metabolic syndrome, *HR* Heart rate, *BMI* Body mass index, *SBP* Systolic blood pressure, *DBP* Diastolic blood pressure, *FPG* Fasting plasma glucose, *TC* Total cholesterol, *TG* Triglyceride, *HDL-C* High density lipoprotein cholesterol, *LDL-C* Low density lipoprotein cholesterol

^*^Comparison between the male and female groups

### The correlation between the number of ideal CVH metrics and the risk of abnormal glucose metabolism

After adjustment for age, sex, occupation, family history of diabetes, heart rate, smoking, drinking, and other factors, people with ideal CVH metrics 3, 4, 5–6 had a significantly lower risk of abnormal glucose metabolism than those with onlyideal CVH metrics 0–2. The results of the trend test showed that each increase in an ideal CVH metric was associated with a 23% reduction in the risk of IFG and a 20% reduction in the risk of T2DM, with ORs (95% CI) of 0.77 (0.65–0.92) and 0.80 (0.71–0.90), respectively (*P*- trend < 0.001) (Table 2).

**Table 2:** The relationship between the number of ideal CVH metrics and the risk of abnormal glucose metabolism

Number of ideal CVH metrics	Total (n)	Prevalence rate (%)		OR (95%CI)^a^	
		IFG	T2DM	IFG	T2DM
0–2	228	10.3	23.7	1	1
3	620	11.5	18.7	0.77 (0.46–1.23)	0.59 (0.42–0.83)^#^
4	720	7.9	19.6	0.49 (0.29–0.83)^#^	0.61 (0.44–0.85)^#^
5–6	416	8.8	15.6	0.56 (0.32–0.99)^#^	0.46 (0.31–0.68)^#^
*P*- trend				< 0.001	< 0.001
For every additional one				0.77 (0.65–0.92)^#^	0.80 (0.71–0.90)^#^

*T2DM* 2 type 2 diabetes mellitus, *IFG* Impaired fasting glucose, *CVH* Cardiovascular health, *OR* Odds ratio, *CI* Confidence interval

^#^*P* < 0.05

^a^Factors adjusted included age, sex, occupation, family history of diabetes, heart rate, smoking and alcohol consumption

### The mediating effect of ideal CVH metrics and T2DMmediated by TyG

The difference in the total effect (path c) between the ideal CVH metrics and the prevalence of T2DM was statistically significant (OR = 0.88, 95% CI: 0.78–0.99), the difference in the direct effect (path c') was not statistically significant (OR = 0.99,95% CI: 0.87–1.23), and the difference in the indirect effect (path ab#, mediating effect) was statistically significant (OR = 0.88,95%CI: 0.84–0.91), that is, TyG fully mediated the number of ideal CVH metrics and the incidence of T2DM. Specific information is presented in Table 3.

**Table 3:** Mediating effect of TyG in the relationship between the number of ideal CVH metrics and the incidence of T2DM

Paths in the mediation model	β value (95%CI)^a^	OR value (95%CI)^a^
the total effect -path c	-0.13 (-0.25– -0.01)	0.88 (0.78–0.99)
the direct effect -path c'	-0.01 (-0.14– 0.12)	0.99 (0.87–1.23)
Path a	-0.12 (-0.15– -0.09)	—
Path b	1.02 (0.83–1.21)	2.77 (2.29–3.35)
The indirect effect -path ab	-0.13 (-0.17– -0.09)	0.88 (0.84–0.91)

*TyG* Triglyceride glucose index, CVH Cardiovascular health, *T2DM* Type 2 diabetes mellitus

^a^Factors adjusted included age, sex, occupation, family history of diabetes, heart rate, smoking and alcohol consumption

## Discussion

This study showed that the prevalence of abnormal glucose metabolism in the elderly was as high as 26.7%, among which IFG and T2DM were 9.5% and 19.0%, respectively. The level of ideal CVH in the elderly was low, and only 57.3% of the population had the number of ideal CVH metrics exceeding 50%. The ideal CVH behavior is a protective factor for abnormal glucose metabolism, and each increase in the ideal CVH metrics can reduce the risk of IFG and T2DM by 23% and 20%, respectively (*P*- trend < 0.001), and TyG, an indicator of insulin resistance, completely mediated the CVH level and the incidence of T2DM.

In general, the prevalence rate of abnormal glucose metabolism has increased [1]. In 2021, the International Diabetes Federation (IDF) estimated that the absolute number of people with diabetes in China would exceed 141 million in 2030 [9]. In 2018, the prevalence of diabetes in elderly people over 60 years of age in China has exceeded 20% [10], and in 2019, people with diabetes in the elderly population over 65 years of age in China represented 25% of global elderly diabetes patients [2], ranking first in the world. For prediabetes, 31 provinces of mainland China with nationally representative cross-sectional data from 2015 to 2017 showed that the weighted prevalence of prediabetes was 35.2% (33.5% to 37.0%) among adults living in China [10]. Meanwhile, evidence published in 2021 shows that the prevalence of IGH was 16% in NHES-Thailand [11]. These evidences are more severe than the results obtained in this study, possibly due to effective prevention and control measures, which have truly reduced the incidence of abnormal glucose metabolism, or the premature death of the elderly population has led to a reduction in the prevalence rate. Additionally, define whether abnormal glucose metabolism only uses FPG, which may miss some patients with abnormal glucose metabolism. Nevertheless, the results of this study showed that the prevalence of abnormal glucose metabolism in the elderly population in this region has been as high as 26.7%. So, early prevention, early detection, and early treatment of abnormal glucose metabolism in the elderly are urgent.

With the aim of improving cardiovascular health in the population, the American Heart Association (AHA) conceptualized ‘ideal cardiovascular health (CVH)’ in 2010. In 2018, a meta-analysis of 88 global studies that included this concept showed that the rate of poor CVH (≤ 2 ideal CVH metrics) in the population was 32.2% and the rate of ideal one (≥ 5 ideal CVH metrics) was only 19.6% [12]. This study showed that the ideal CVH level rate in the elderly population was consistent with the global level, but the overall rate was low, only 20.9%. Previous studies have shown a decrease in cardiovascular events and all-cause mortality in the population with increasing CVH levels [5, 7]. Meanwhile, a 2019 cohort study that included 7758 study subjects showed that compared to subjects with 0–1 ideal CVH metrics, subjects with 2–3 and 4–6 had a 30% and 71% lower risk of T2DM, respectively [13]. This study showed that for each additional ideal CVH metric in this elderly population, the risk of IFG and T2DM decreased by 23% and 20%, respectively, with OR (95%CI) of 0.77(0.65–0.92) and 0.80(0.71–0.90), respectively (*P*- trend < 0.001), meaning that the risk of abnormal glucose metabolism in this population gradually decreased with increasing CVH level, which was consistent with global results. However, based on the analysis of individual components of the CVH index, only the ideal healthy diet and the ideal BMI were protective factors for T2DM, the ideal TC and the ideal blood pressure were protective factors for IFG (Table S1). In high-risk HbA1c-defined prediabetes, additional measurement of FPG will add little to the evaluation of cardiometabolic risk [14], only systolic BP and LDL-c were weakly associated with FPG levels, subjects exhibited significant variation in cardiometabolic risk factors according to their FPG levels but this variation was generally accounted for by age, gender, adiposity or ethnicity. Although this study used CVH as a composite index and did not specifically analyze the relationship between each of these components and abnormal glucose metabolism, it showed that the risk of abnormal glucose metabolism decreases as the number of normal in the CVH index increases.

The results of the mediation effect analysis suggested that in the older population, TyG played a complete mediation role in the reduction of the risk of T2DM risk by the effect of the ideal CVH level, suggesting that the TyG index can be used as an early marker to detect the high-risk group of abnormal glucose metabolism in the older population, during early prevention and early detection of abnormal glucose metabolism. Furthermore, there are some researches showing that low HDL-c is associated with prediabetes and predicts progression to diabetes, especially in the Asian population [11, 15]. The present study did not have an in-depth analysis in this direction limited to the characteristics of the independent variables, whether the effect of HDL-c reduction on the risk of developing glycemic abnormalities is the same as that of a reduction in the number of CVH indicators should be explored in further studies.

This study has several limitations. Firstly, the study population is all 65 years or older in China. Although the evidence is more relevant to guiding health interventions for the elderly, the extrapolation of the findings to other ages or geographical groups and the interpretation of causal associations will be somewhat limited. This is mainly due to differences in the distribution of body fat and lifestyle habits from the other geographical groups. Meanwhile, compared to other age groups, there is also the obesity paradox in the elderly population. Secondly, 1984 participants were included in this cross-sectional study; although the sample size can meet the statistical efficacy of this study, the stability and causal relationship of its findings still needs to be verified by a large sample of studies. Finally, due to age or other physical reasons, some people could not be included in this study due to the absence of critical variables, although measures of expanded sample size were applied in the design phase and multivariate adjustment in the statistical analysis phase, which could still partially bias the study findings.

## Conclusion

Abnormal glucose metabolism has become one of the major chronic disease burdens in the elderly. Taking into account the limitations of the effectiveness of a single CVH indicator and the synergistic effect of multiple indicators, when discussing potential strategies to improve CVH and reduce the risk of abnormal glucose metabolism in the elderly population, we should not have a negative attitude towards improving a single indicator, but focus on improving comprehensive indicators. The increase in CVH level may effectively reduce the risk of abnormal glucose metabolism. Effectively increasing the ideal CVH level will be one of the important ways to reduce the incidence of glucose metabolism abnormalities in the elderly, and one of the important ways to solve the problem of standardizing the management of glucose metabolism abnormalities in the elderly.

### Supplementary Information

**Additional file 1: Table S1.** The individual components of the CVH index and their specific impacts on abnormal glucose metabolism.

## Data Availability

The datasets used and/or analysed during the current study available from the corresponding author on reasonable request.
