# Supplementary material for: Association between ideal cardiovascular health and abnormal glucose metabolism in the elderly: evidence based on real-world data
Source: BMC Geriatr. 2024 May 10;24:414. doi: 10.1186/s12877-023-04632-4 (PMC11084128; doi:10.1186/s12877-023-04632-4)
Supplement: Supplementary file 1 — Additional file 1: Table S1. The individual components of the CVH index and their specific impacts on abnormal glucose metabolism. [file 12877_2023_4632_MOESM1_ESM.docx]

| Table S1. The individual components of the CVH index and their specific impacts on abnormal glucose metabolism. | | | | | |
| --- | --- | --- | --- | --- | --- |
|  | T2DM | |  | IFG | |
|  | OR (95%CI) | *P* |  | OR (95%CI) | *P* |
| Ideal healthy diet | 0.494 (0.245,0.996) | **0.049** |  | 0.379 (0.118,1.216) | 0.103 |
| Ideal physical activity | 0.960 (0.750,1.280) | 0.743 |  | 0.811 (0.568,1.158) | 0.249 |
| Ideal smoking status | 1.022 (0.639,1.634) | 0.928 |  | 0.506 (0.202,1.263) | 0.144 |
| Ideal BMI | 0.729 (0.576,0.923) | **0.009** |  | 0.784 (0.555,1.108) | 0.169 |
| Ideal TC | 0.932 (0.745,1.167) | 0.541 |  | 0.468 (0329,0.666) | **<0.001** |
| Ideal blood pressure | 0.930 (0.741,1.167) | 0.530 |  | 0.561 (0.394,0.800) | **<0.001** |
| IFG: impaired fasting glucose; T2DM: type 2 diabetes mellitus; BMI: body mass index; TC: total cholesterol; OR: odds ratio; CI: confidence interval. | | | | | |
